# Cloning of human *ABCB11 g*ene in *E*. *coli* required the removal of an intragenic Pribnow-Schaller Box before it’s Insertion into genomic safe harbor AAVS1 site using CRISPR–Cas9

**DOI:** 10.12688/f1000research.26659.1

**Published:** 2020-12-23

**Authors:** Nisha Vats, Madhusudana Girija Sanal, Senthil Kumar Venugopal, Pankaj Taneja, Shiv Kumar Sarin

**Affiliations:** 1 Department of Molecular and Cellular Medicine, Institute of Liver and Biliary Sciences, New Delhi, Delhi, 110070, India; 2 Department of Life Sciences, South Asian University, New Delhi, Delhi, 100021, India; 3 Department of Biotechnology, Sharda University, Noida, Uttar Pradesh, 201310, India; 4 Department of Hepatology, Institute of Liver and Biliary Sciences, New Delhi, Delhi, 110070, India

**Keywords:** Progressive Familial Intra Hepatic Cholestasis Type-2, CRISPR-Cas9, AAVS1 site, human ABCB11/BSEP, MsbA, E. Coli, Endotoxin, Lipid A transporter, Pribnow-Schaller Box, cloning

## Abstract

**Background:** Genomic safe harbors are sites in the genome which are safe for gene insertion such that the inserted gene will function properly, and the disruption of the genomic location doesn’t cause any foreseeable risk to the host. The AAVS1 site is the genetic location which is disrupted upon integration of adeno associated virus (AAV) and is considered a ‘safe-harbor’ in human genome because about one-third of humans are infected with AAV and so far there is no apodictic evidence that AAV is pathogenic or disruption of AAVS1 causes any disease in man.  Therefore, we chose to target the AAVS1 site for the insertion of *ABCB11*, a bile acid transporter which is defective in progressive familial intra hepatic cholestasis type-2 (PFIC-2), a lethal disease of children where cytotoxic bile salts accumulate inside hepatocytes killing them and eventually the patient.

**Methods**: We used the CRISPR Cas9 a genome editing system to insert the *ABCB11* gene at AAVS1 site in human cell-lines.

**Results:** We found that human *ABCB11* sequence has a “Pribnow- Schaller Box” which allows its expression in bacteria and expression of ABCB11 protein which is toxic to *E*. *coli*; the removal of this was required for successful cloning. We inserted *ABCB11* at AAVS1 site in HEK 293T using CRISPR-Cas9 tool.  We also found that the ABCB11 protein has similarity with *E*. *coli *endotoxin (lipid A) transporter MsbA.

**Conclusions:** We inserted *ABCB11* at AAVS1 site using CRISPR-Cas9; however, the frequency of homologous recombination was very low for this approach to be successful *in vivo*.

## Introduction

**Figure 1.**
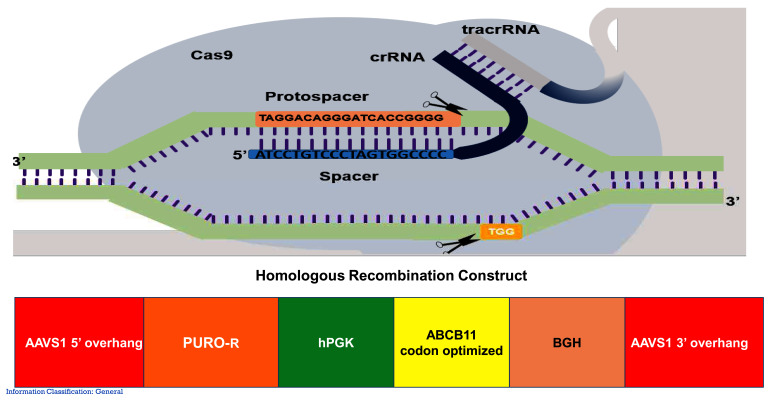
The Cas9-sgRNAs designed and the recombination cassette (donor vector). The gene of interest ( *ABCB11*) was flanked by 5’ and 3’ overhangs which are homologous to the AAVS1 site in chromosome 19. *ABCB11* gene was driven by human promoter phospho-glycerol kinase.

Progressive familial intrahepatic cholestasis type-2 (PFIC2), a severe liver disease which is familial, neonatal, progressive and often fatal which results from a mutation of ATP binding cassette subfamily B member 11 ( *ABCB11*) gene which codes for an ABC transporter bile salt export pump (BSEP) ^1, 2^. Mutations in *ABCB11* gene results in accumulation of cytotoxic taurocholate and other cholate conjugates leading to progressive hepatocyte destruction ^1^. Currently the definitive cure for PFIC2 is liver transplantation, which is limited by suitable donor organs. Gene therapy, allogenic hepatocyte transplantation ^3^ and autologous transplantation of hepatocytes/liver organoids differentiated from ‘gene corrected’ induced pluripotent cells could be future options ^4, 5^. Adeno-associated virus (AAV) is so ubiquitous in man and animals that about 30% of the world population are positive for this virus and to date no disease is proven to be associated with this virus ^6– 9^. In our study we inserted *ABCB11* gene at the AAVS1 site using CRISPR-Cas9 tool ( Figure 1) in HEK293T cells and a fibroblast line.

## Methods

### Cloning of *ABCB11*

We PCR-amplified the 3966 bp *ABCB11* (from cDNA prepared from total RNA of human liver tissue) using multiple overlapping primers ( *Extended data*, Supp. Table 1) ^10^ which were assembled by overlap extension PCR ^11^. Phusion DNA polymerase (NEB, US Cat. #M0530L) was used as per the manufacturers protocol. Annealing temperate for all PCRs unless otherwise stated was 60°C C for 20s and an extension time 30s/kb at 72°C. Initial denaturation was carried out at 98°C for 30s and 5s in subsequent steps. In general, we used 24 cycles to amplify PCR products for cloning (and 32 cycles for other PCRs). We used 1.0 unit of the enzyme per 50 µl PCR reaction. The product was cloned in ‘donor’ vector having AAVS1 recombination overhangs on both ends and ampicillin resistance for selection ( *Extended data*, Supp. Figure 1) ^10^. The upstream (5’) overhang sequence (803 bp) in the vector was homologous to a sequence (NCBI Sequence ID: NC_000019.10, 55115768 to 55116570) inside the *PPP1R12C* gene ( Figure 1), which would be in-frame with a 2A ribosome skip sequence and puromycin resistance gene if insertion of the construct happens by homologous recombination with the target site. This is region was followed by a poly adenylation signal and the downstream (3’) overhang sequence which was the continuation of the 5’ overhang (837 bp). We inserted the ABCB11 sequence driven by human phospho-glycerol kinase (hPGK) promoter between these overhang sequences. DH5α cells, JM 109 and One Shot™ Stbl3™ Chemically Competent *E. coli* cells (ThermoFisher Catalog#:C737303) were used for transformation and cloning.

### Bioinformatics

Bacterial promoter prediction was done using BPROM, a prediction tool for bacterial promoters ^12^. DNA or protein sequence comparisons were done using the appropriate tool from NCBI BLAST platform ^13^. Primers were designed manually or using NCBI Primer Blast ^14^. 

### gRNA

We designed two guide RNAs targeting the AAVS1 site ( Figure 1) and cloned in two vectors expressing SPCas9 and SACas9 at BsaI sites ( *Extended data*, Supp. Figure 2) ^10^ following the standard gRNA design, cloning protocols and resources ^15^. Off-target analysis was done using the tool Custom Alt-R® CRISPR-Cas9 guide RNA ^16^.

### Cell culture

HEK293T, HepG2 and FS1 (fibroblast) cells were grown in high glucose DMEM (Hi-Media Lab, Mumbai, Cat.# AL111-500ML) supplemented with 10% fetal bovine serum (CellClone, Genetix Biotech Asia, New Delhi, Cat.# CCS-500-SA-U), 1x penicillin (100U/ml) and streptomycin (100 µg/ml) (Hi-Media, Mumbai Cat. # A018-5X100ML). When 80% confluent, the cell lines were transfected with Cas9-sgRNA vectors (without the donor vector). At 48 h post-transfection, about 10000 of these cells were used for comet assay ^17^ and genomic DNA (gDNA) isolated from the remaining cells was used for T7-endonuclease assay ^18^ to evaluate the *in vitro* ‘DNA cutting’ activity of Cas9-sgRNA construct. Subsequently, we transfected these cells with the donor vector, Cas9-sgRNA vector and a control vector (pEGFPN1) in the ratio: 2:1:1 using PEI ^19^ from Sigma Aldrich, Inc. (CAS #9002-98-6). After 48 h the cells were imaged and used for downstream applications. Half of the transfected dishes were serially passaged without puromycin selection for two weeks and DNA and protein were isolated. On the remaining dishes puromycin selection was started following the manufacturer’s protocol ^20^ after 36 h of transfection and puromycin resistant colonies at 8 µg/mL were further cultured in puromycin containing media and gDNA was isolated.

### Western blot

Whole-cell extracts (see *Cell culture*), scraped out and extracted using RIPA Lysis and Extraction Buffer, were run on 10% SDS-PAGE and transferred to a polyvinylidene difluoride membrane using a transfer apparatus following the standard protocols (Bio-Rad). After incubation with 5% nonfat milk in TBST (10 mM Tris, pH 8.0, 150 mM NaCl, 0.5% Tween 20) for 60 min, the membrane was washed once with TBST and incubated with rabbit antibodies against human ABCB11 (Affinity, Catalog #DF 9278) 1: 2000 dilution; human β-actin (Santa Cruz Cat.# SC4778), dilution 1:1000; 4°C overnight. The membrane was washed with TBST buffer and incubated with a 1:5000 dilution of horseradish peroxidase-conjugated anti-rabbit (Santa Cruz Cat# SC-2004)/anti-mouse antibodies (Cat.#SC-2005) for 2 h at room temperature. Blots were washed with TBST four times and developed with the ECL system (Bio-Rad, US Cat.#170-5060) according to the manufacturer’s protocols. Raw, uncropped images from western blotting are available as Underlying data ^21^.

### T7 Endo Nuclease Assay

T7 endo I assay detects heteroduplex DNA that results from annealing DNA stands that have been modified after a sgRNA/Cas9 mediated cut to DNA strands without modifications. T7 Endonuclease-1 was purchased from NEB, US (Cat. #NEB #E3321) and was used to digest the PCR products amplified from gDNA extracted from Cas9-sgRNA transfected (test) and un-transfected cells (control) using primers flanking the expected Cas9-sgRNA cut sites following the manufactures protocol ^18^. PCR gel images are available as *Underlying data* ^21^.

### Comet assay

A total of 50–100 cells treated as described in *Cell culture* were embedded in 0.7% low-melting agarose and mounted on a precoated slide and was immersed in alkaline 0.1% SDS solution overnight, neutralized and electrophoresis was done in an alkaline buffer (pH 10) at 0.74 V/ cm for 30 minutes ^17^. Comet assay images are available as *Underlying data* ^21^.

### Sequencing

Sequencing of PCR products and plasmids were conducted by Invitrogen Bioservices India Pvt. Ltd., a part of Thermo Fisher Scientific, Whitefield, Bengaluru, PIN 560 066, India and Medauxin, Bengaluru, AMCO Colony, Koti Hosahalli, Bengaluru, PIN 560 092, India.

## Results

### Human *ABCB11* gene/product is toxic to *E. coli* cells

**Figure 2.**
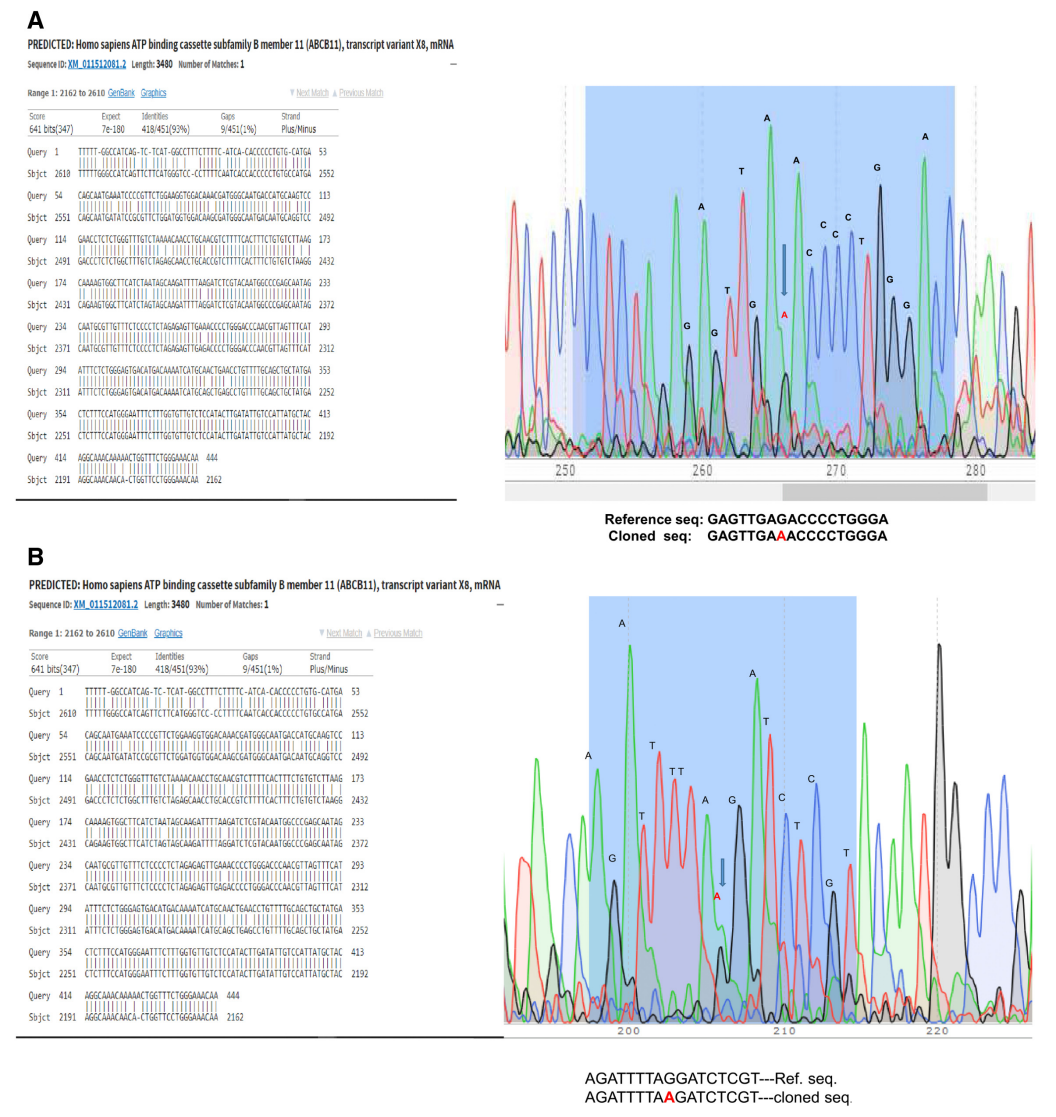
The *ABCB11* gene cloned into *E. coli* showed mutations despite multiple cloning attempts. ( **a**) G to A mutation is marked for example. ( **b**) In another example, sequence of another clone, mutation was in a different site.

Few ampicillin resistant DH5α *E. coli* colonies which we got after transformation were screened for the insert by colony PCR. One colony was positive for all the fragments of the *ABCB11* gene. Sequencing revealed that mutations in ABCB11 ( Figure 2). Repeated attempts failed and we considered the possibility of unstable DNA sequences. Therefore, we tried JM109 which gave one positive colony and plasmid was isolated. However, after we soon found the bacteria failing to grow or losing the plasmid on subsequent cultures. Therefore, we transformed One Shot™ Stbl3™ Chemically Competent *E. coli* cells, which are suitable for cloning unstable DNA segments. We got many positive colonies, however, upon overnight culture the bacteria formed a big pellet (partly lysed bacteria) which cannot be resuspended in phosphate buffered saline. Therefore, we concluded that the *ABCB11* gene/gene product is toxic to bacterial cells. It is possible the ABCB11, being a membrane transporter, may be toxic to bacteria. Sequencing files are available as *Underlying data* ^21^.

### Identification and removal of a Pribnow-Schaller Box in human ABCB11 for cloning in *E. coli*

**Figure 3.**
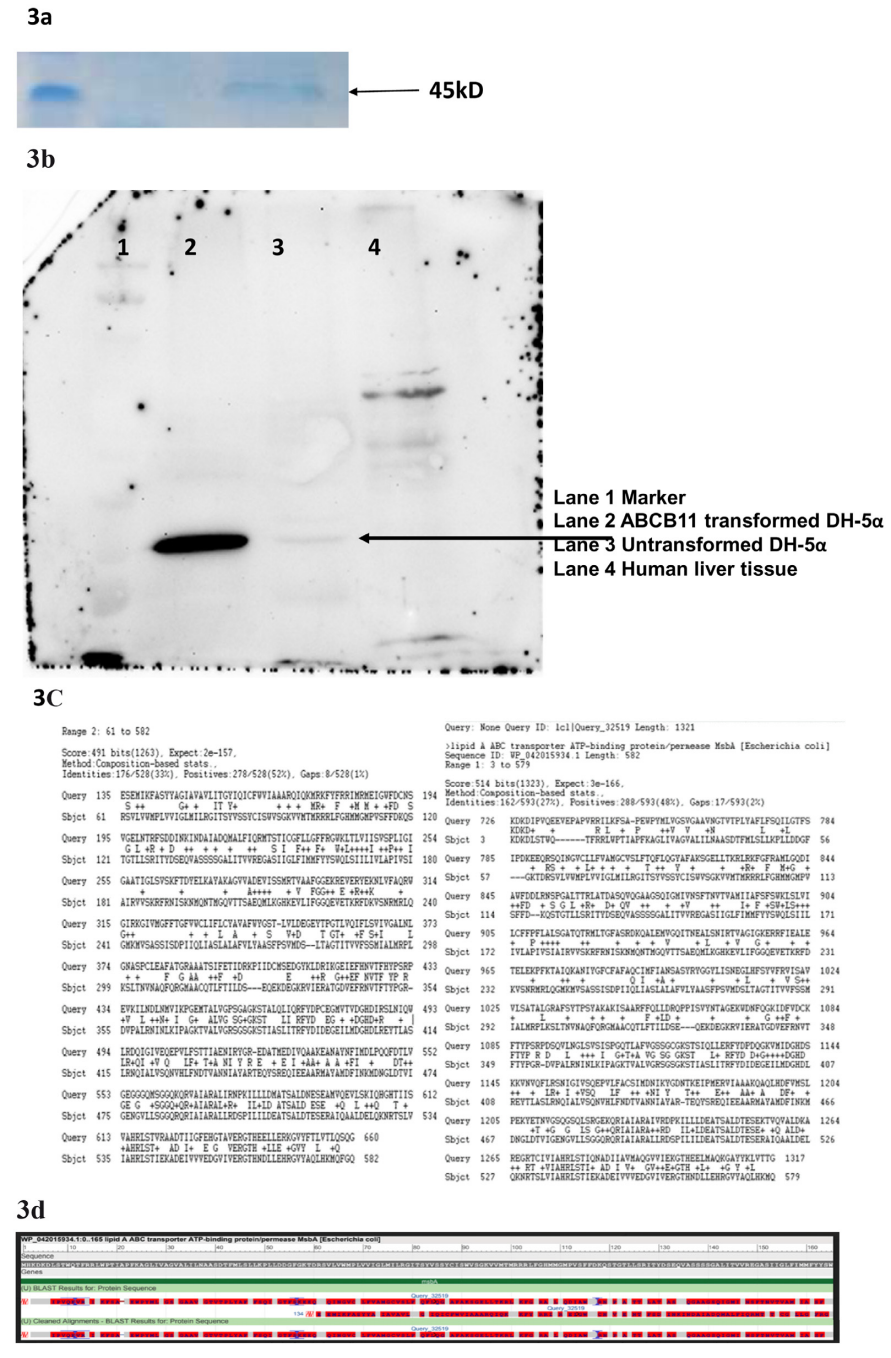
( **a**) Total protein extract from *E. coli* transformed the donor vector on Poly Acryl amide Gel Electrophoresis followed by Coomassie blue staining showed differential expression multiple proteins. ( **b**) Western Blot with anti-human ABCB11 antibody shows multiple bands including one probably corresponding to MsbA- a bacterial Lipid A transporter. ( **c**) A bioinformatic analysis (Protein BLAST) revealed ABCB11 and MsbA are sharing conserved domains. ( **d**) Sequence alignment of *ABCB11* and MsbA showing conserved domains.

**Figure 4.**
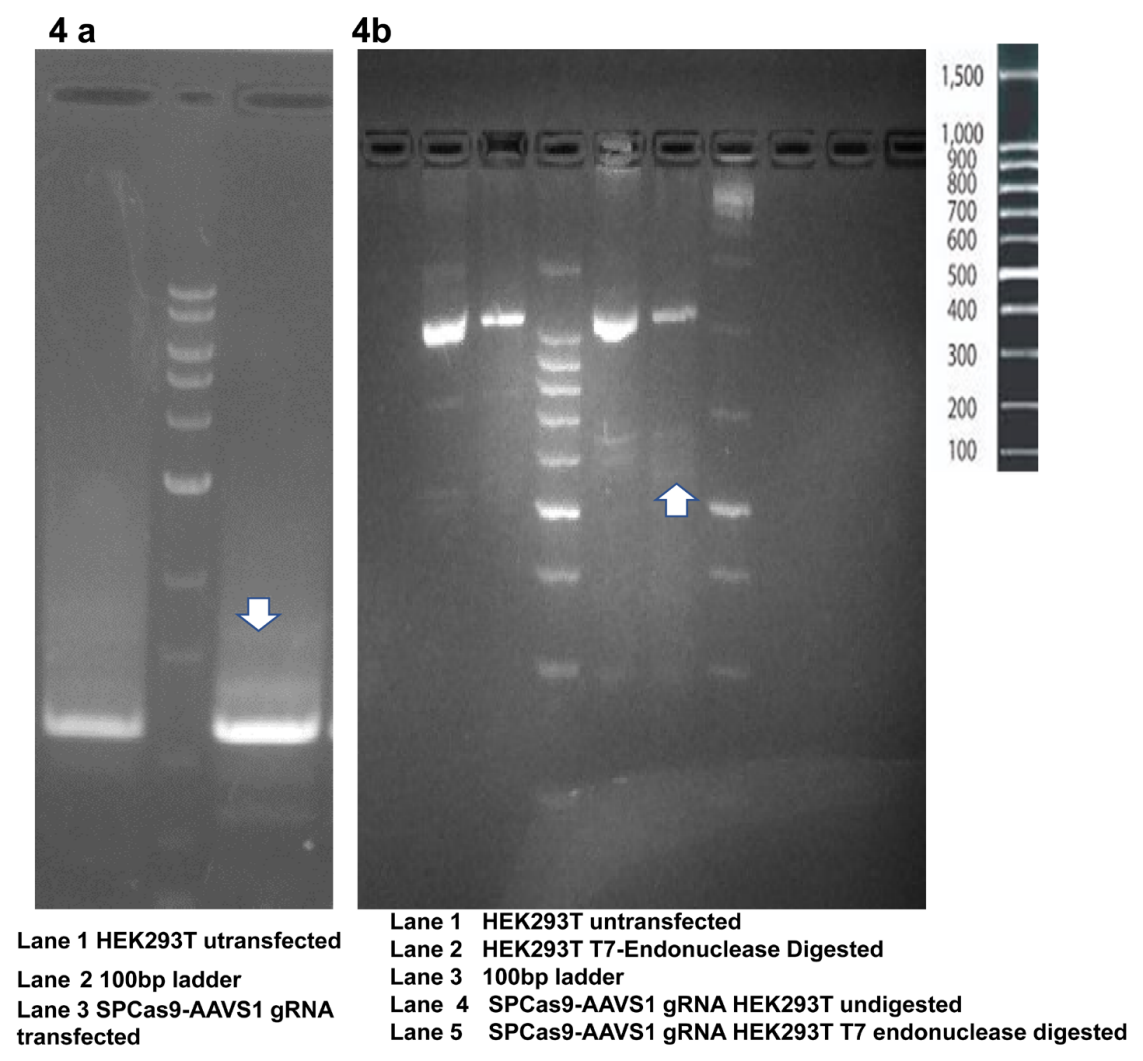
Agarose gel electrophoresis of PCR amplified products. **4a** The PCR product (even without T7 digestion) shows a distinct band pattern resulting from the formation of heteroduplex. **4b**. PCR amplified product after digestion with T7 endonuclease shows faint bands resulting from the digestion of heteroduplexes.

We conducted PAGE followed by Coomassie staining to see differential protein expression between ABCB11 donor vector transformed bacteria versus untransformed bacteria ( Figure 3a; *Extended Data*) ^21^. We found differential expression of a few proteins. We subsequently performed a western blot and interestingly antibody against human ABCB11 identified a specific protein over expressed in the transformed bacterial cells ( Figure 4b). However, in our construct ABCB11 gene was under a eukaryotic promoter. Considering the possibility of some DNA elements which have similarity to bacterial promoters inside the ABCB11 sequence we performed a bioinformatic analysis using BPROM to predict hidden bacterial promoters ( *Extended data*, Supp. Table 2) ^10^. The promoter-site (Pribnow-Schaller box tca **tataat**) containing sequence (ggttttgagtcagataaatca **tataat**aat) which we identified was modified to (ggtTTCGAAtcagataaatcaTACAACaat) by PCR using an oligonucleotide primer sequence incorporating the modified sequence and subsequent overlap extension PCR amplification of the entire gene fragment. With this modification, we were able to clone *ABCB11* coding sequence which was not toxic to bacteria. 

### ABCB11 is similar to *E. coli* lipid A (endotoxin) transporter MsbA

A protein BLAST search identified MsbA (UniProtKB - P60752), a member of the ABC transporter superfamily. MsbA, a 64.46 kD protein, has an important role in *E. coli* Lipid A (endotoxin or LPS) transport ( Figure 3c, d). This protein flips core endotoxin from its site of synthesis on the inner leaflet of the inner membrane to the outer leaflet of the inner membrane. western blot showed identified a unique band in the donor vector transformed *E. coli* while the untransformed *E. coli* also showed a faint band but specific band at the same position ( Figure 3b).

### Verification of the gDNA cutting activity of Cas9-sgRNA construct

**Figure 5.**
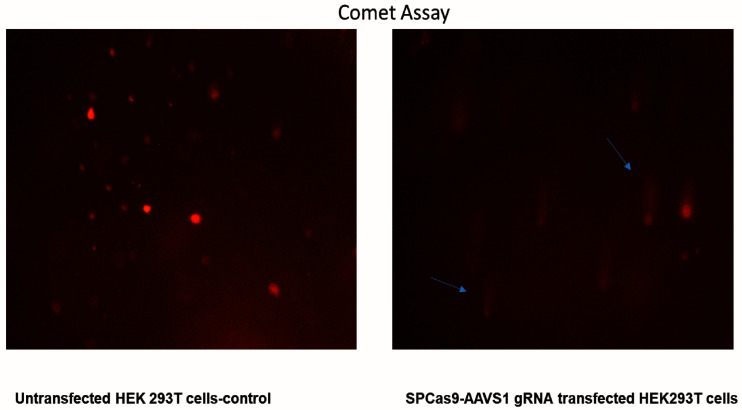
Cas9-sgRNA cause DNA damage, revealed by a Comet assay. The untreated cells have intact round/oval nuclei while the Cas9-sgRNA treated cells shows a comet shaped nucleus because of DNA damage.

T7 Endonuclease Assay and Comet Assay were used to evaluate the gDNA cutting activity of Cas9-sgRNA. We observed digestion of heteroduplexes at the CRISPR-Cas9 cut sites which were sensitive to T7 endonuclease ( Figure 4a). These heteroduplexes were observed on the agarose gel electrophoresis of PCR products as well ( Figure 4b). The Cas9-sgRNA damaged the genome of the transfected cells leading to the formation of comet shaped nuclear material upon electrophoresis ( Figure 5).

### Off-target analysis

Oligonucleotide PCR primers were designed for bioinformatically predicted off-targets ( *Extended data*, Supp. Tables 3a, b) ^10^. We amplified these segments using genomic DNA extracted from the treated cells (48 h post-transfection with SPCas9-sgRNA vector) as template. The PCR products were sequenced and analyzed for sequence disruption ( Table 1). 

**Table 1.** Selected off targets for crRNA GGGGCCACTAGGGACAGGAT (PAM TGG). Oligonucleotide primers designed to amplify these off-target sites to verify off target disruptions.

Off-Targets	Genes which may be affected	Primers	Size (bp)	Disruption
chr10: +119439168	G protein-coupled receptor kinase 5	F-AGCCTCCATCCAGATCCTGT	655	No
		R-TGGCCACAGTGTGTTTCCT		
chr6: +36797686	copine-5 isoform e	F-TCCCAGTCTGCCCTCTCTTT	716	No
		R-CATGCCTCACTGCTCTCACA		
chr12- GRCh38.p13	MMP17 flanks 3’	F-GGGTCTCTGCTCTGGAAACC	791	Yes
		R-TGGGAGATCTTGGGAGAGGG		
chr18: +8749293	microtubule cross-linking factor 1	F-GCCGTCAAAATGGCACACAT	630	No
		R-TTGGATCTGCCCAAGCTCAG		
chr20-GRCh38.p13	TMEPAI isoform d flanks 5’	F-ATCAGTCTGTCGCTACCCCT	471	No
		R-ATGAGGGGTCAGCCTATGGT		
chr9-GRCh38.p13	Di-Ras2 flanks 3’ PRO21346 flanks 5’	F-CCTCTTGCCTTGCTGCTTTG	455	No
		R-TTGCTGTCTGACAACCTCCT		

### Validation of ABCB11 expression in a fibroblast cell line

**Figure 6.**
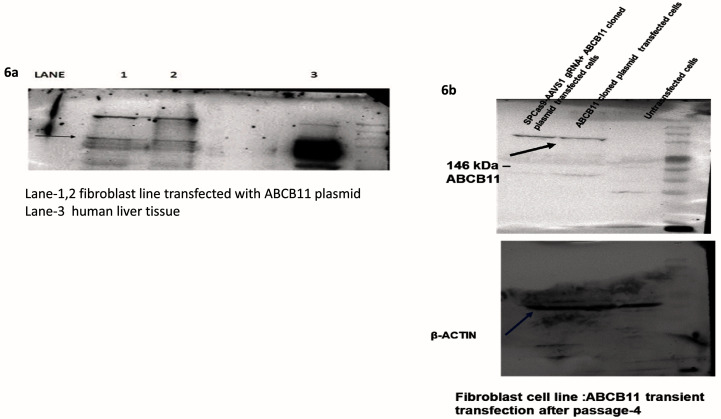
( **a**) Western Blot with anti-human ABCB11 antibody confirmed the expression after transient transfection with the donor vector having *ABCB11*. ( **b**) Western Blot was done using total protein isolate from fibroblasts (which does not naturally express *ABCB11*) after four passages post-co-transfection of the donor vector with *ABCB11* gene and the CRISPR-Cas9-sgRNA vector.

Western blotting done with total protein extract of fibroblasts 48 h post-transfection with the *ABCB11* donor vector showed the expression of ABCB11 protein ( Figure 6a). A fibroblast line was used in these experiments because they don’t express ABCB11, naturally while cell lines such as HEK293T and liver cell lines such as HepG2 do express ABCB11.

Western blotting was repeated with total protein extract from fibroblasts after 2 weeks (fourth passage) post-transfection with SPCas9-sgRNA vector together with the donor vector containing *ABCB11*. This blot also showed the expression of ABCB11 protein ( Figure 6b) suggesting the integration of *ABCB11* into the host genome.

### PCR amplification and sequencing to verify the integration of ABCB11 to AAVS1 site

**Figure 7.**
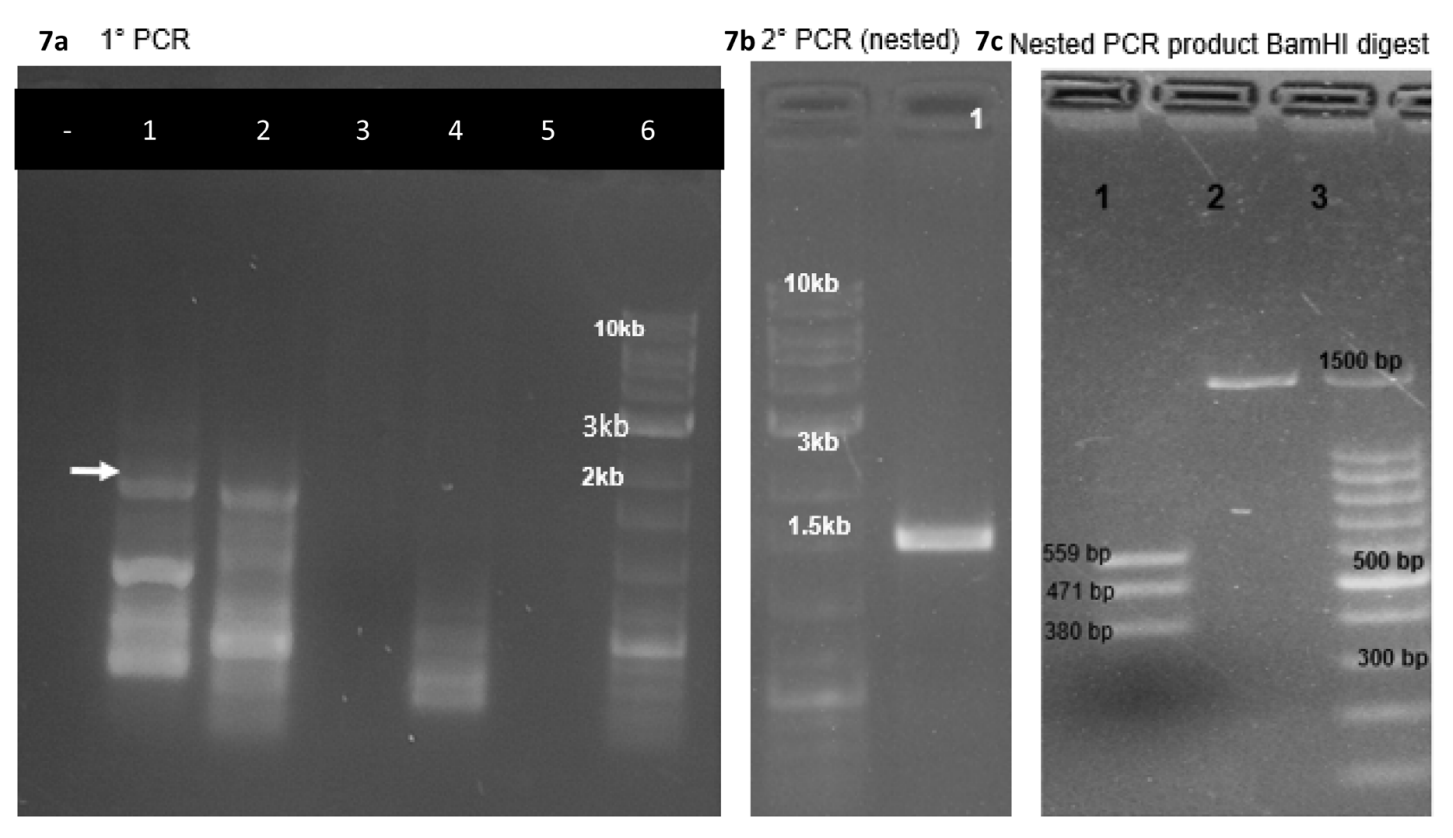
( **a**) The gDNA isolated from transfected cells (Cas9-sgRNA plasmid alone) after 72h, (Cas9-sgRNA plasmid plus donor vector) after 21 days of puromycin selection were used as PCR templates (lane 1, 2) with a forward primer complementary to a region upstream of the 5’ recombination overhang of the vector and a reverse primer complementary to a sequence in the puromycin resistance gene to amplify a segment spanning from a site in the host cell genomic DNA slightly upstream of the genomic integration site to a segment donated by the donor vector (puromycin resistance gene). Note that genomic DNA from untreated HEK293T cells did not give any products in the expected range (lane 3, 4). ( **b**) The PCR product mentioned in ( **a**) was used as a template for a nested PCR. ( **c**) The PCR product mentioned in ( **b**) was confirmed by restriction enzyme digestion (BamH1) and sequencing (Primers: Extended data, Supp.Table 4) ^10^.

**Figure 8.**
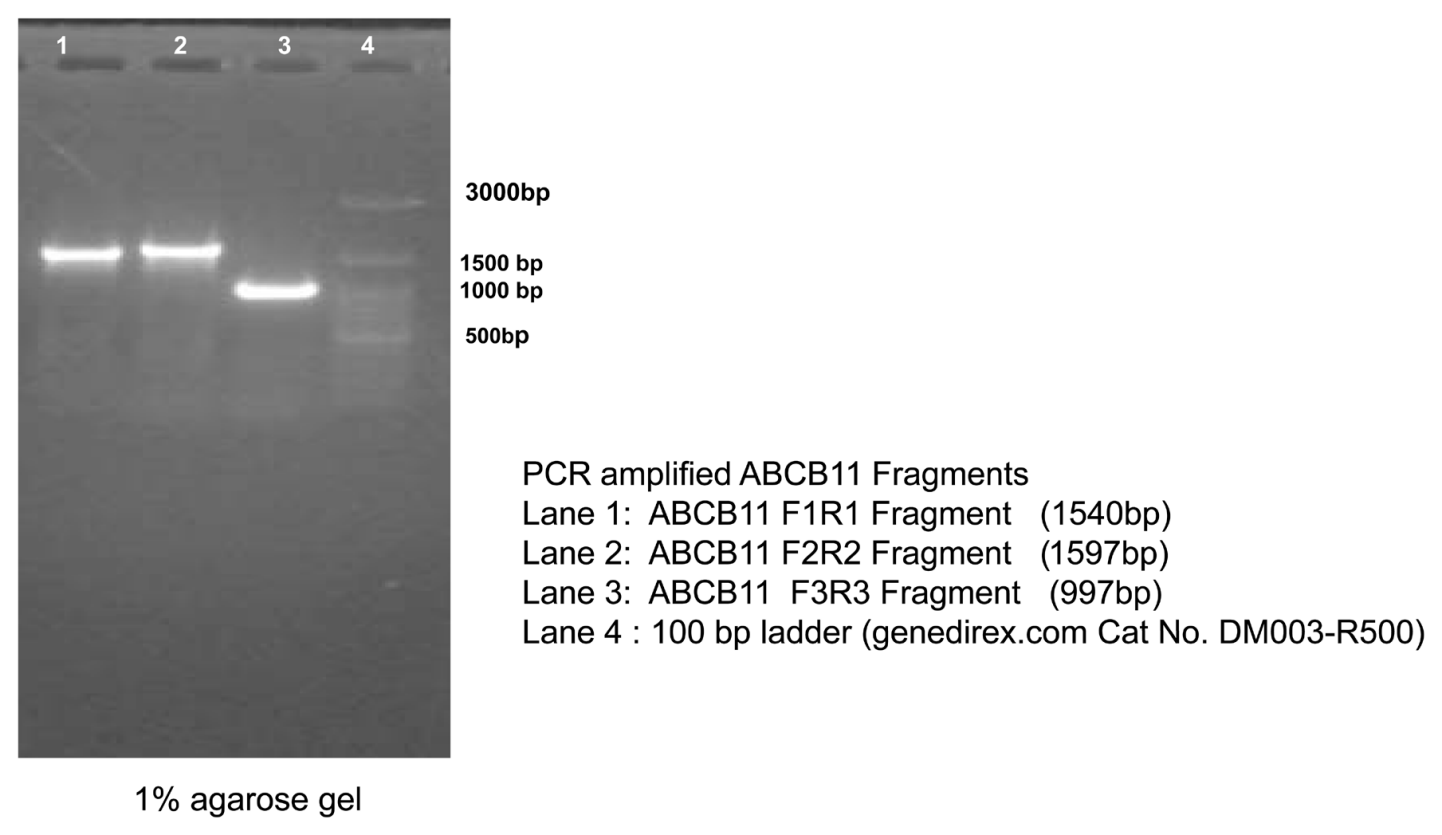
PCR products amplified from the genomic DNA. *ABCB11*-specific primers were used, which would give specific PCR products from the inserted cassette. This was done to make sure the *ABCB11* gene was not deleted from the cassette integrated to the host cell genome. The PCR products showed the expected sizes confirming that the amplified products originated from the integrated cassette and not from the native *ABCB11* gene present in the cell line.

We obtained only three puromycin resistant colonies upon transfecting about 20 million HEK cells with a transfection efficiency of 70 to 80% in four 6 cm dishes. ****The gDNA isolated from transfected cells (Cas9-sgRNA plasmid alone) after 72h, (Cas9-sgRNA plasmid plus donor vector) after 21 days of puromycin selection were used as PCR templates with a forward primer complementary to a region upstream of the 5’ recombination overhang of the vector and a reverse primer complementary to a sequence in the puromycin resistance gene to amplify a segment spanning from a site in the host cell genomic DNA slightly upstream of the genomic integration site to a segment donated by the donor vector (puromycin resistance gene). This PCR product was used as a template for a nested PCR and product was confirmed by restriction enzyme digestion (BamH1) and sequencing (Primers: *Extended data*, Supp. Table 4 ^10^, Figure 7). We also PCR-amplified parts of ABCB11 using primers ( *Extended data*, Supp. Table 1) ^10^ which would give specific PCR products from the inserted cassette, to make sure the gene is not deleted from the cassette integrated to the host cell. PCR products showed the expected sizes confirming that the amplified products are from the cassette and not from the native ABCB11 gene present in the cell line ( Figure 8). 

## Discussion

To our knowledge, this is the first time the *ABCB11* gene was inserted into the AAVS1 safe-harbor using CRISPR-Cas9 technology in human cells. Liver directed gene therapy is another approach and was successful in rodents ^22^. Adeno associated vectors do not integrate and therefore the effect of gene therapy many not last in human beings, especially in infants, as the viral vector dilutes out as the cells proliferate in a growing liver ^23^. Another approach is transplantation of hepatocytes differentiated from gene corrected patient iPSC ^4, 24, 25^. AAVS1 site is considered as a ‘safe haven’ in human genome ^26^ where we chose to insert the gene mutated in PFIC. We could not find any other study which attempted to insert the *ABCB11* gene at the AAVS1 site. AAV is a common virus and it is considered non-pathogenic because the seroprevalence of wild-type AAV in humans ranges from 40% for AAV8 to 70% for AAV1 and AAV2, yet; we are not aware of any disease caused by AAV ^6, 7, 27^. AAV integration into AAVS1 site causes disruption of PPP1R12C (protein phosphatase 1 regulatory subunit 12C). However, this gene is not associated with any disease ^27^. A puromycin gene was placed in the donor cassette such that the puromycin gene will be transcribed only if homologous recombination happens. We obtained only a few puromycin resistant colonies suggesting that homologous recombination was a rare event (~10 ^-7^). This suggests that *in vivo* gene therapy using CRISPR-Cas9 technology making use of homology directed gene repair could be difficult. “Targeted Integration and high transgene expression in AAVS1 Transgenic mice after *in-vivo* hematopoietic stem cell transduction with HDAd5/35++ Vectors” ^28^ is reported; however, to achieve this they used an adenoviral gene delivery system with AAV5 ITRs and AAV35 helper. Integration of AAV/ Cas9 into Cas9 mediated cut sites is a potentially hazardous consequence of this approach ^29, 30^.

**Figure 9.**
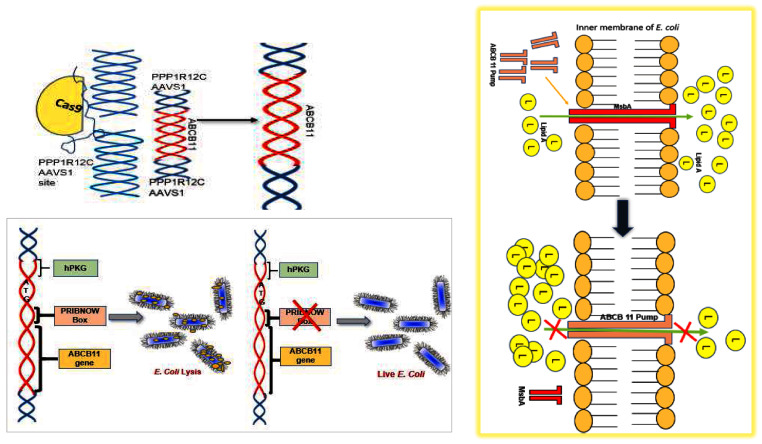
The *ABCB11* gene (which codes the transporter of human bile salts) is targeted to AAVS1 site using a construct which has 5’ and 3’ overhangs which are homologous to the AAVS1 site. A Pribnow box was detected inside *ABCB11*, which allowed the gene to transcribe in *E. coli*, causing bacterial lysis, probably through competitive replacement of a homologous transporter protein in *E. coli* ( *E. coli* Endotoxin (Lipid A) Transporter) MsbA, resulting in Lipid A (L) accumulation inside the bacteria.

We found that the human *ABCB11* donor vector transformed bacteria either died or the *ABCB11* gene sequence got mutated meaning either the DNA sequence or the ABCB11 protein has some untoward effects on bacteria. We performed a western blot and found that in ABCB11-transformed bacterial clones giving a band around 45 kD and HepG2 cells/liver tissue is giving a band at around 140 kD which corresponds to ABCB11. It was interesting to note that untransformed *E. coli* cells are also showing a band although very faint around 45 kD which suggested the possibility of a bacterial protein which might have structural similarity to human ABCB11. We performed a bioinformatic search and identified MsbA an *E*. *coli* protein which functions as a lipid transporter (~64 kD). MsbA is involved in the transport of bacterial endotoxin-a function like the ABCB11 which transports the bile salts which are lipid derivatives ^31, 32^. Another interesting observation was the identification of a bacterial promoter sequence (Pribnow-Schaller Box) in human *ABCB11* causing unexpected expression of ABCB11 protein and bacterial toxicity ( Figure 9). This was an important lesson for us because we spent a lot of time trying to clone ABCB11. It is therefore important to search for and eliminate if any bacterial promoter sequences or similar elements are identified, for successful cloning of eukaryotic/toxic genes in *E. coli*. We do not know how exactly ABCB11 caused bacterial toxicity. Possibly, expression of ABCB11 in *E. coli* might be destabilizing the *E. coli* membrane, since ABCB11, being a lipid transporter, is a membrane-spanning protein.

Alternatively, the human protein, which is similar to the *E. coli* protein might have caused a competition between the bacterial transporter MsbA and human protein for membrane incorporation resulting in the accumulation of endotoxin within *E. coli* cells because unlike the bacterial transporter the membrane incorporated ABCB11 might not be able to transport endotoxin out. This raises the possibility that endotoxin is toxic to *E. coli* itself if it is not exported and MsbA can therefore be considered as a drug target. This point required further validation ( Figure 9). The advantage of such an antibiotic is that it will be selective to endotoxin producing microbes. More research is required in this direction. It may be noted that endotoxin producing microbes play an important role in sepsis ^33^ and diseases such as non-cirrhotic portal hypertension ^34– 36^.

To conclude, we successfully inserted *ABCB11* at the AAVS1 site using CRISPR-Cas9, however, the frequency of homologous recombination was very low as evident from the number of puromycin resistant colonies. With this low efficiency, with the current technology it is unlikely that this approach would be successful in *in-vivo* gene editing. It is worth, exploring MsBA as a novel antibiotic target for LPS producing bacteria, although our data in this direction is primitive and requires further validation. 

## Data availability

### Underlying data

Harvard Dataverse: Sequencing Data, supplementary data to Cloning of Human ABCB11 Gene in E. coli required the removal of an Intragenic Pribnow-Schaller Box before it’s Insertion into Genomic Safe Harbor AAVS1 Site using CRISPR Cas9. https://doi.org/10.7910/DVN/32TXCD ^21^.

This project contains the following underlying data:

- 2020_06_20_211607 actin.jpg. (Uncropped western blot image.)
- 4a .T7 endonuclease digestion.jpg. (PCR gel image.)
- 4b. PCR without T7 endonuclease.jpg (PCR gel image.)
- 4c... Cas9-sgRNA treated cells comet assay.jpg. (Image taken from Comet assay, treated cells.)
- 4c...... untreated cells comet-1.jpg. (Image taken from Comet assay, untreated cells.)
- ABCB11_fibroblasts.jpg. (Uncropped western blot image.)
- ABCB11_fragments.tif. (PCR gel image.)
- ABCB11_WB_P4_2020_06_19_183004-1.tif. (Uncropped western blot image.)
- DH5a WB-ABCB11-long_Exposure.jpg. (Uncropped western blot image.)
- DH5a WB-ABCB11.jpg. (Uncropped western blot image.)
- E.Coli_PAGE_Coumasse.jpg. (Uncropped PAGE gel.)
- JM109_ABCB11 WB.tif. (Uncropped western blot image.)
- nested PCR product BamH1 digest.jpg. (PCR gel image.)
- Nested PCR secondary.jpg. (PCR gel image.)
- Nested_Primary pcr.jpg. (PCR gel image.)
- Repeat_WB_2020_07_11_181831.jpg. (Uncropped western blot image.)
- Sequencing data.7z. (Sequencing data produced in the present study.)
- T7endo 30420202.jpg. (PCR gel image.)

### Extended data

Harvard Dataverse: Supplementary Tables to Cloning of Human ABCB11 Gene in E. coli required the removal of an Intragenic Pribnow-Schaller Box before it’s Insertion into Genomic Safe Harbor AAVS1 Site using CRISPR Cas9. https://doi.org/10.7910/DVN/NTUOXM ^10^.

This project contains the following extended data:

- Supp-Tables-revised-HepInt.docx. (Supp. Tables 1–4.)
- SuppFigures.pptx. (Supp. Figure 1a, b.)
