# Supplementary material for: Cloning of human ABCB11 gene in E. coli required the removal of an intragenic Pribnow-Schaller Box before it’s Insertion into genomic safe harbor AAVS1 site using CRISPR–Cas9
Source: F1000Res. 2020 Dec 23;9:1498. [Version 1] doi: 10.12688/f1000research.26659.1 (PMC8030117; doi:10.12688/f1000research.26659.1)
Supplement: Peer review history

# Peer review: Reviewer response for version 1

**DOI:** 10.5256/f1000research.29430.r81735

**Published:** 2021-04-07

**Authors:** Nagendra Chaturvedi

**Affiliations:** 1 Department of Pediatrics, Hematology, and Oncology, University of Nebraska Medical Center, Omaha, NE, USA

The manuscript is well-designed with a valuable contribution to the successful cloning of ABCB11, a key defective gene in PFIC2.

 I only have a few minor comments:

- Is that common to see mutations in ABCB11 gene while expressing it in the prokaryotic or eukaryotic expression system? 
- Do authors know the functional consequences if this gene is not mutated in the expression system? 
- Title of manuscript is too long. 

Is the work clearly and accurately presented and does it cite the current literature?

Yes

If applicable, is the statistical analysis and its interpretation appropriate?

Yes

Are all the source data underlying the results available to ensure full reproducibility?

Yes

Is the study design appropriate and is the work technically sound?

Yes

Are the conclusions drawn adequately supported by the results?

Yes

Are sufficient details of methods and analysis provided to allow replication by others?

Yes

Reviewer Expertise:

Molecular Cell Biology

I confirm that I have read this submission and believe that I have an appropriate level of expertise to confirm that it is of an acceptable scientific standard.

# Peer review: Reviewer response for version 1

**DOI:** 10.5256/f1000research.29430.r81026

**Published:** 2021-04-06

**Authors:** Mitradas Panicker

**Affiliations:** 1 Department of Physiology and Biophysics, School of Medicine, University of California, Irvine, CA, USA

This is an interesting and useful observation regarding the cloning of the human ABCB11 gene in *E.coli* prior to its original goal i.e. inserting ABCB11 into the AASV1 safe harbor.

 The aim was to insert the ABCB11 gene into human cells to establish the initial steps for gene therapy in liver. The authors found that they could not clone their construct of the gene in E. coli. Careful observation and analysis led them to a Pribnow-Schaller Box within the sequence. They proved their hypothesis by modifying the Box and successfully cloning the gene in *E. Coli. *The work is detailed and carefully done and also suggests further areas such as the potential use of MsbA as a drug target.

Comments:

 The title could be corrected to reflect the result 'proper' since the removal of the Pribnow-Schaller box is not a necessary requirement for insertion into the AASV1 locus. It became a necessity due the cloning method that had to be adopted.

 The manuscript requires some additional editing for clarity. 

 Figure 1 would benefit by indicating the site of the 2A insertion, the hPGK sequence should be marked as a promoter.

 It is not clear what is the ABCB11 codon optimization for and also what it was optimized for as noted in Fig.1.

 It would be more accessible if the Pribnow-Schaller Box and the sequences changed are depicted as a figure than as a supplementary table and a few lines of text. If text is preferred then the sequence numbers of the nucleotides involved and the reference sequence should be provided in the text.

 The observation that homologous insertion was obtained at very low frequency does not necessarily preclude the strategy from being used 'in vivo' since the frequency would be highly dependent on the strategy and the sequences chosen. For e.g. a homology-independent strategy with same construct might prove fruitful. Perhaps the discussion could reflect this.

 The supplementary Figure of the Donor Vector does not show the ABCB11 sequence. This should also be addressed.

 The observation by Chang and Gray should be more clearly emphasized in the Discussion.

 MsbA as a drug target should also refer Zhang *et al.* (2018) ^1^.

Is the work clearly and accurately presented and does it cite the current literature?

Yes

If applicable, is the statistical analysis and its interpretation appropriate?

Not applicable

Are all the source data underlying the results available to ensure full reproducibility?

Yes

Is the study design appropriate and is the work technically sound?

Yes

Are the conclusions drawn adequately supported by the results?

Yes

Are sufficient details of methods and analysis provided to allow replication by others?

Yes

Reviewer Expertise:

Molecular Neurobiology, Stem Cells, Neuroscience, Cell Biology

I confirm that I have read this submission and believe that I have an appropriate level of expertise to confirm that it is of an acceptable scientific standard.

## References

[1] : Cell-based screen for discovering lipopolysaccharide biogenesis inhibitors. *Proceedings of the National Academy of Sciences*.2018;115(26) : 10.1073/pnas.1804670115 6834-6839 10.1073/pnas.1804670115 PMC604206529735709
